# Advancement of humanized mice and leading applications in immunological disease models

**DOI:** 10.1186/s41232-026-00427-3

**Published:** 2026-06-05

**Authors:** Satoru Munakata, Yuki Imura, Sota Fujimori, Ryota Sato, Piruzyan Mariam, Yuzo Koda

**Affiliations:** Research Unit, Research Division, Tanabe Pharma Corporation, Yokohama, Kanagawa 227-0033 Japan

**Keywords:** Humanized mice, Autoimmune diseases, Disease models, Immune reconstitution

## Abstract

Disease models are used to evaluate drug candidates, and compounds that are highly effective in vivo models have traditionally been prioritized for development. While conventional ‘gold standard’ animal models have been central to autoimmune drug discovery, there is increasing recognition that addressing unmet medical needs requires models capable of capturing patient pathophysiology beyond the scope of these classical systems. Accordingly, models that reflect human disease mechanisms not reproducible in conventional animals are becoming increasingly important. Humanized mice are immunodeficient mice transplanted with human immune cells, hepatocytes, thymic tissue, and other components to create a human-like biological environment that cannot be replicated in wild-type mice. Research on humanized mice has advanced through efforts to reconstitute a diverse human immune system in mice, together with accumulating knowledge of patient-specific factors such as autoantibodies and autoreactive T cells. Additionally, single-cell analyses and human tissue studies are underway to recreate the human-specific disease phenomena in humanized mice. In this review, immune-system-humanized mice are used to provide a comprehensive overview of recent advances in immune-system-humanized mouse technologies, their applications to immune-related disease models, and their current utilization in drug discovery research.

## Background

The field of autoimmune disease drug discovery research has increasingly recognized the importance of disease mechanisms and patient pathophysiology that cannot be fully captured by conventional “gold standard” animal models, particularly to address unmet medical needs [1, 2]. Even within the same autoimmune disease, patients often exhibit different immune phenotypes and pathological presentations; thus, each patient’s complex pathology should be understood and reflected in appropriate models [3, 4]. Molecularly targeted therapies such as anti‑tumor necrosis factor-α (TNF-α) antibodies and Janus kinase (JAK) inhibitors, originally identified using classical animal models, have had a major impact on the treatment of autoimmune diseases, and their clinical value is unquestionable [5–8]. However, it has become clear that conventional animal models alone have limited ability to predict therapeutic responses across the heterogeneous patient populations seen in autoimmune disorders. For example, anti‑TNF-α antibodies show robust and reproducible anti‑inflammatory effects in the collagen‑induced arthritis (CIA) model, yet in rheumatoid arthritis patients, approximately 20–30% exhibit primary non‑response and an additional 10–20% per year develop secondary loss of response [9, 10]. Similarly, while anti‑TNF-α antibodies are highly effective in the dextran sulfate sodium (DSS)-induced colitis model, the placebo‑adjusted clinical response rate in ulcerative colitis at week 8 is limited to approximately 32–40%, as shown in clinical trials [11]. Furthermore, although anti‑interleukin‑12/23 (IL‑12/23, p40) antibodies tend to outperform anti-IL-17A (interleukin-17A) antibodies in mouse psoriasis models, clinical psoriasis shows the opposite pattern, with IL‑17A inhibitors achieving substantially higher PASI 90 (Psoriasis Area and Severity Index 90) response rates than ustekinumab (anti-human IL-12/23 p40 neutralizing antibody) [12–14]. Together, these discrepancies highlight the need for disease models that more accurately reflect human immune pathophysiology and inter‑patient heterogeneity. Instead of evaluating patient-specific markers or histopathological features, comprehensive datasets, including transcriptomic and proteomic analyses, are increasingly being incorporated into humanized mouse studies [15–17]. Furthermore, whether the distinctive cell subsets identified in patients by single-cell RNA sequencing or spatial transcriptomics are reproduced in in vitro and in vivo models should be verified [18, 19]. For example, peripheral helper (Tph) cells, follicular helper (Tfh) cells, and tissue-specific Treg cells differ significantly between humans and mice [20–22]. Thus, reproducing these human-specific cells in models has become a key challenge. Recent technological advances have increased the value of sophisticated in vitro evaluation systems, such as organoid cultures and organ-on-a-chip devices [1, 2]. Despite advances in sophisticated in vitro systems, in vivo studies in rodents or larger animals remain essential for assessing efficacy, pharmacokinetics, and safety. Accordingly, this review focuses on humanized mice and summarizes their major types, immune-related disease models, and applications in drug discovery. Specifically, we discuss classical immune-system-humanized mice generated by engrafting immunodeficient mice with either human CD34⁺ hematopoietic stem cells (hHSCs) or human peripheral blood mononuclear cells (hPBMCs), as well as improved immune-system-humanized mice refined by removing specific donor cell subsets or by genetic modification of the host. Human liver-chimeric (liver-humanized) mice created by transplanting human hepatocytes and bone marrow-liver-thymus (BLT) mice established by the simultaneous transplantation of human bone marrow, liver, and thymus tissue fragments are also discussed. In addition, we present examples of immune-related disease models developed using immune-system- humanized mice and highlight their current use in evaluating therapeutic candidates (e.g., antibody drugs and protein therapeutics) that cannot be adequately assessed in conventional mouse models. This review comprehensively summarizes the immunological characteristics of various humanized mouse models, incorporating the latest findings, and provides an in-depth discussion that extends to their applications in drug discovery. In addition, it offers clear guidance on the selection and use of appropriate humanized mouse models according to specific research objectives.

## Overview of various humanized mouse models

### hHSC transplantation model

The hHSC transplantation model is a humanized mouse created by transplanting human CD34⁺ HSCs into immunodeficient mice (such as NSG, NOG, NRG, or BRG strains), enabling long-term reconstitution of human hematopoietic cells in the bone marrow, thymus, and peripheral tissues [23–25]. Post-transplantation, human hematopoietic cells engraft and expand over time in multiple tissues (including peripheral blood, spleen, bone marrow, liver, and lung), and mature human T cells selected in the mouse thymus are supplied to the periphery [25, 26]. Compared with the hPBMC model (described in the following section), this approach generates a broader spectrum of immune cell lineages, including T cells, B cells, natural killer (NK) cells, monocytes, and granulocytes, thereby enabling a more comprehensive reconstitution of the human immune system [27, 28]. Additionally, because positive and negative selection of human T cells occurs in the mouse thymus, the incidence of graft-versus-host disease (GVHD) is markedly lower, with symptoms appearing around 16–24 weeks, which is a significant advantage unique to the hHSC model [29, 30]. Conversely, reconstitution of the human immune system usually takes about 10–16 weeks, which is a practical disadvantage compared with the hPBMC model [31]. Transplantation conditions significantly influence the degree and balance of human immune reconstitution. For example, the proportion of human CD45⁺ cells in peripheral blood can reach approximately 70% at 12 weeks after transplantation, although donor-dependent variability has also been observed. Indeed, the frequency of repopulating HSCs among umbilical cord blood-derived CD34⁺ cells has been reported to differ by up to 10-fold between donors [31]. Moreover, the initial engraftment rate of human CD45⁺ cells injected into neonatal mice (immediately after birth) via the intrahepatic or facial vein is significantly higher than that of cells injected into adult mice via the tail vein [27, 31, 32]. The source of donor hHSCs also affects the reconstitution pattern: umbilical cord blood-derived hHSCs tend to engraft well in the early phase, fetal liver-derived hHSCs initiate T-cell development earlier, and adult bone marrow-derived hHSCs yield lymphoid-to-myeloid ratios closer to those of adult humans [23, 33]. Furthermore, differences in the host mouse strain affect outcomes: NSG/NOG strains typically allow stable initial engraftment and tissue distribution, whereas the C57BL/6-Rag2-null IL2Rγ-null (B6RG) strain has a longer lifespan, making it suitable for long-term observation [34]. Functionally, because T-cell education in the thymus occurs via mouse major histocompatibility complex (MHC), human leukocyte antigen (HLA)-restricted antigen-specific T-cell responses (particularly CD8⁺ T cells) are weak [35]. Germinal center formation is insufficient, and the production levels of class-switched Immunoglobulin G (IgG) are low. Human dendritic cells exhibit a low capacity for cross-presentation. Human neutrophils have reduced chemotaxis and activity. Mouse red blood cells are scarcely replaced by human red blood cells [27, 36]. Therefore, additional improvements, such as knock-in (KI) of human cytokine genes or the introduction of human HLA genes, have been attempted to enhance functionality (described in the following section), but challenges remain. In summary, the hHSC model enables long-term reconstitution of multi-lineage human immune cells and allows tracking of peripheral immune cell changes. By optimizing preconditioning (e.g., busulfan treatment), neonatal injection routes, and donor hHSC source selection, engraftment levels and the balance of immune cell subsets can be improved [37]. However, issues, such as weak HLA-restricted T-cell responses, poor long-term humoral immune responses, and incomplete replacement of erythroid and myeloid cells, remain, and these are expected to be improved by genetically modified humanized mice.

### hPBMC model

The hPBMC model enables rapid reconstitution of a human T-cell-dominant immune system by injecting hPBMCs into immunodeficient mice (such as NSG, NOG, or BRG strains). Within 1–2 weeks post-injection, human CD45⁺ cells appear in the peripheral blood, the majority of which are CD3⁺ T cells [25, 28, 38, 39]. In particular, CD4⁺ memory T cells become predominant, whereas B cells and monocytes/dendritic cells are detected only at low frequencies, and few human red blood cells or granulocytes are generated. Thus, this model enables efficient in vivo analysis of human T-cell-dependent immune responses and has been widely used to evaluate T-cell responses in tumor immunology and infectious diseases. However, a significant limitation is that within 2–3 weeks after PBMC transfer, human T-cell-mediated GVHD develops, making long-term observation difficult [40–42]. The causes of GVHD are thought to include human T cells recognizing and attacking mouse tissue antigens and host innate immune responses directed toward eliminating human cells. The liver is the organ most consistently affected by early damage and infiltration [41]. In addition to the liver, the lung is one of the first organs injured after PBMC transfer. Cytokines, such as TNF-α and interferon gamma (IFN-γ), are thought to contribute to the onset of GVHD [42]. The propensity for GVHD also varies with the mouse strain background. For example, in NOD-background mice, a polymorphism in the Sirpa gene allows mouse SIRPα to bind strongly to human CD47, delivering a “don’t-eat-me” signal that favors human cell engraftment [43]. In contrast, B6RG-background mice exhibit a weaker CD47-SIRPα interaction, leading to more rapid phagocytosis of human cells; however, in Rag2-null Il2rg-null CD47-null triple-knockout mice, phagocytosis of human cells is further suppressed, prolonging engraftment and the observation window [44]. In NOG mice, the absence of CD11c⁺B220⁺CD122⁺ cells has mitigated xenorejection responses [45]. Additionally, removing CD8⁺ T cells or CD4⁺ T cells from PBMC fraction can alleviate GVHD symptoms [46]. Furthermore, knocking out the host mouse MHC class I and II molecules prevents human CD8⁺ and CD4⁺ T cells from recognizing mouse MHC in lymphoid organs, thereby attenuating GVHD in peripheral tissues. This indicates that human T-cell responses via host MHC molecules are a major driver of GVHD in humanized mouse models [47, 48]. As illustrated by these findings, the host mouse’s genetic background and genetic modifications can extend the engraftment period in the hPBMC model and modulate human T-cell persistence and tissue infiltration. Moreover, the PBMC donor characteristics also influence the strength and phenotype of the immune response. For instance, cord blood-derived PBMCs have a higher proportion of naive T cells, whereas adult donor PBMCs contain more memory T cells, leading to differences in reactivity and GVHD manifestation. Preconditioning of the mice (e.g., irradiation or busulfan treatment) is less critical than in the hHSC model, though low-dose irradiation has been reported to increase initial engraftment modestly [49]. In cancer immunology research, the hPBMC model has been used in studies in which patient-derived tumor fragments or tumor cell lines are co-implanted orthotopically or ectopically with PBMCs to evaluate human T-cell cytotoxicity in the tumor microenvironment and responses to immune checkpoint inhibitors [50–52]. In infectious disease research, transferring PBMCs harboring chronic viruses (such as human immunodeficiency virus; HIV) into mice has recapitulated phenomena observed in humans, including CD4⁺ T-cell depletion, upregulation of activation markers, and induction of virus-specific responses [38, 53]. Conversely, this model shows poor human B-cell engraftment and persistence and, even with antigen immunization, still fails to generate class-switched IgG or germinal centers, making it unsuitable for evaluating long-term humoral immunity [25, 28, 38, 39, 41, 42]. In summary, the hPBMC model enables rapid observation of human T-cell-centric immune responses and is particularly useful for testing interventions that affect in vivo human T-cell activity or GVHD pathology.

Additionally, integrated multi-omic approaches are increasingly applied to hHSC models to gain deeper mechanistic insights. Lu et al. used single-cell transcriptomic and epigenomic profiling to identify a previously unrecognized “homeostatically matured” human cDC2 subset (DC2hm) in hHSC-NSG mice. This subset upregulates tolerance-associated genes, including tissue-restricted self-antigens, and is driven by a TSLP-dependent program that enables efficient induction of FOXP3⁺ regulatory T cells. This study exemplifies how comprehensive multi-omics can uncover human-specific immune regulatory programs, such as mechanisms of peripheral tolerance, beyond conventional phenotypic analyses, thereby enhancing the mechanistic value of hHSC-based humanized mouse models [54].

### CD8TΔhPBMC transplantation model

**Fig. 1:**
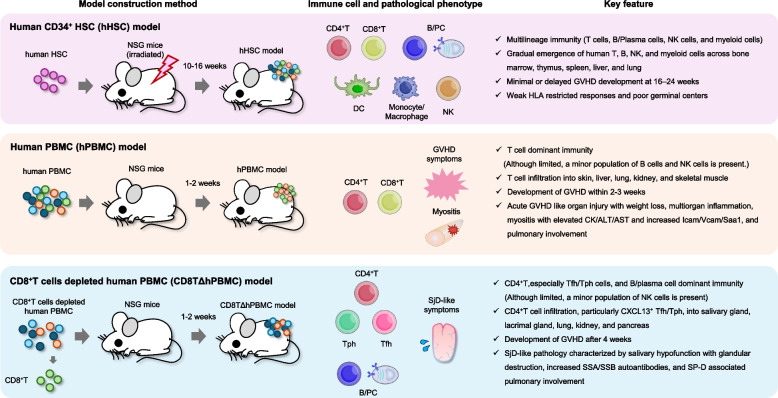
Characteristics of humanized immune system mouse models. The characteristics of three humanized immune system mouse models that serve as the foundation for disease modeling are shown. The human hematopoietic stem cell transplantation (hHSC) model enables long-term multilineage human immune reconstruction with minimal or delayed graft-versus-host disease (GVHD) and can reproduce diverse immune cell subsets [27, 29, 30]. However, it exhibits weak human leukocyte antigen (HLA)-restricted responses, poor germinal center activity, and a longer time to model establishment [31, 35]. The human peripheral blood mononuclear cell (hPBMC) model rapidly develops severe T-cell-driven GVHD with multiorgan inflammation, including dermatitis, myositis, and pulmonary involvement, within 2–3 weeks [25, 28, 38, 39]. This model is suitable for evaluating activated human T cells and GVHD, but observations are limited by early GVHD onset, which makes assessing other cell types difficult [40–42]. The CD8⁺ T-cell-depleted human PBMC transplantation model (CD8TΔhPBMC model) offers significant advantages over the conventional hPBMC model [55]. By depleting CD8⁺ T cells, this model suppresses GVHD symptoms while promoting Tfh/Tph-cell expansion and B-cell proliferation. Consequently, it exhibits Sjögren’s disease-like pathology, including salivary gland destruction, autoantibody production, and lung involvement [55]. This improvement enables researchers to investigate Tfh/Tph- and B-cell biology, as well as SjD pathology, without the confounding effects of GVHD. However, the time to GVHD onset remains a limiting factor, and it remains challenging to observe CD8⁺ T cells and other immune cell subsets in this model [55]

Our group recently proposed a CD8TΔhPBMC transplantation model (CD8TΔhPBMC model), in which transferred PBMCs are depleted of CD8⁺ T cells, as a new humanized mouse model with an immune phenotype distinct from those of the hHSC and hPBMC models [55]. In this model, the absence of CD8⁺ T cells dramatically reduces the typical GVHD observed in the standard hPBMC model. In the conventional hPBMC model, starting around 2–3 weeks post-transfer, mice exhibit significant weight loss, decreased activity, dermatitis, and tissue damage in organs such as the liver and lungs [55]. In contrast, the CD8TΔhPBMC model shows markedly reduced weight loss, and GVHD onset can be delayed by over a month. Moreover, removing CD8⁺ T cells fundamentally alters the pattern of human immune cell reconstitution in vivo. Most notably, human CD4⁺ T cells engraft and expand dominantly, with a striking skew toward differentiation into Tfh and Tph cells. Concurrently, human B and plasma cell engraftment and activation in the spleen are enhanced, enabling in vivo production of donor-derived autoantibodies [55]. Indeed, at 1 month after the transfer of CD8⁺ T cell-depleted PBMCs, approximately 1 × 10⁸ human CD45⁺ cells were engrafted in the spleen, more than 90% of which were αβ T cells. In addition, whereas only 2–3 × 10⁶ B cells engrafted in conventional hPBMC mice, more than 1 × 10⁷ B cells were detected in this model [55]. This phenotype was discovered serendipitously during a study of idiopathic inflammatory myopathy-like symptoms in a standard hPBMC model, where CD8⁺ T cells were removed to assess their contribution to muscle pathology [56]. High-parameter flow cytometry and single-cell RNA sequencing revealed that IL-21⁺ CXCL13⁺ Tph cells infiltrated tissues, such as the salivary glands, lungs, kidneys, and pancreas, and that prior to GVHD onset, these mice developed Tph-cell-dependent Sjögren’s syndrome-like symptoms (details of this pathology model are described in a later section). This model addresses the limitations of the hHSC model, including the long time required to develop disease phenotypes and the inability to sustain patient-derived antigen–receptor repertoires, and markedly suppresses GVHD, enabling observation of human-specific Tfh/Tph cells and Sjögren’s pathology unconfounded by GVHD. Thus, it provides a useful new platform for studying human Tfh/Tph and B-cell biology as well as for modeling autoimmune diseases such as Sjögren’s disease. Figure 1 illustrates the differences in the characteristics of the hHSC, hPBMC, and CD8TΔhPBMC models as foundational humanized mice.

Although this model represents an improvement over the hPBMC model, the onset of GVHD-like manifestations approximately 4 weeks after cell transfer remains a limitation. Further refinements are therefore required to enable long-term observation of immune mechanisms. In addition, pathological features driven by CD8⁺ T cells cannot be captured. Moreover, compared with the hPBMC model, engraftment of cell populations critical for antibody-production processes, such as B cells and Tfh/Tph cells, can be confirmed; however, the model has not yet recapitulated de novo antigen recognition induced by immunization, including T-cell receptor (TCR) and B-cell receptor (BCR) repertoires rearrangement and in vivo antibody production. To address this issue, we consider that establishing human antigen-presenting cells in vivo, such as dendritic cells, will be crucial.

### Liver-transplanted and BLT models

#### BLT model

The BLT model is generated by implanting human fetal liver and thymic tissue under the renal capsule of immunodeficient mice and transplanting donor‑matched CD34⁺ hHSCs, thereby reconstituting a human immune system [49, 57]. Human T-cell development and selection occur in the thymic graft, enabling HLA-restricted T-cell responses [49, 57]. BLT mice show strong human immune reconstitution in mucosal tissues (e.g., intestine and female reproductive tract); for example, hCD45⁺ engraftment in the small-intestinal lamina propria is ~ 15-fold higher in NSG-BLT mice than in hPBMC models [58], supporting analyses of local infection and early responses in vaginal HIV models [59, 60]. BLT models can also mount antigen-specific humoral responses (e.g., neutralizing HCMV-specific IgM/IgG; improved B-cell maturation and dengue-specific IgM/IgG in NSG-SGM3-BLT) [61, 62]. However, HIV infection can persist despite virus-specific cellular and humoral responses, indicating that BLT-induced immunity may be insufficient for long-term viral control [63]. Overall, BLT mice are widely used to study mucosal/local immunity, antigen-specific IgG responses, and the pathogenesis and protective mechanisms of mucosal infections [49, 59, 61].

#### Human liver-chimeric model

This model involves transplanting human hepatocytes into highly immunodeficient mice, enabling replacement of the mouse liver parenchyma with human hepatocytes [64]. In the Alb-TK-NOG model, for instance, liver-specific toxic injury to mouse hepatocytes creates vacant niches that are efficiently repopulated by human hepatocytes [65, 66]. In such liver-humanized mice, increased human albumin production and human-specific cytochrome P450 activity allow evaluation of human-specific hepatic function and drug metabolism in vivo [65, 66]. In addition, liver-humanized approaches have also been extended to the evaluation of human liver pathology, including fibrosis and metabolic dysfunction associated steatotic liver disease (MASLD). For example, transplantation of human hepatic stellate cells into immunodeficient mice enabled in vivo assessment of human cell-driven fibrogenesis with histological fibrotic changes [67]. More advanced multicellular humanized liver systems incorporating human non-parenchymal cells have reproduced aspects of human liver pathology, including fibrosis and MASLD, in a more human-relevant hepatic microenvironment [68]. However, these models still have limitations, as many remain hepatocyte-centered and do not fully reconstruct the human non-parenchymal and immune microenvironment required to model chronic liver disease with full fidelity [69, 70]. Despite these limitations, human liver-chimeric models provide a valuable platform for translational studies of human-specific drug metabolism, pharmacokinetics/pharmacodynamics (PK/PD), and human cell-driven fibrogenesis in vivo. In addition, when combined with an immune system-humanized model (e.g., an hHSC model), they can also be used to evaluate in vivo changes in drug-metabolizing enzyme expression under inflammatory conditions and liver remodeling [71, 72].

#### Human lung tissue xenograft model

In this approach, human lung tissue fragments are subcutaneously or under the renal capsule implanted into immunodeficient mice, creating chimeric mice that harbor human lung tissue [73]. In studies of SARS-CoV-2 infection, the human lung graft model supported viral RNA replication and infectious virion production within the implanted human lung tissue, with human epithelial cells (hACE2⁺ regions) serving as the infection targets [74, 75]. In this model, infection led to time-dependent increases in human cytokines/chemokines, including IFN-α/β, IL-6, IL-8, TNF-α, monocyte chemoattractant protein-1, and C-X-C motif chemokine ligand 10 [74, 75]. By applying antiviral therapies (e.g., human IFN-α) in this model, one can simultaneously evaluate viral infection dynamics in human lung tissue, the local inflammatory response, and therapeutic efficacy [74, 75].

#### Human intestinal organoid (HIO) xenograft model

This method transplants cultured HIOs under the kidney capsule or into the intestinal wall of immunodeficient mice, reconstructing a tissue comprising human intestinal epithelium and stroma [76, 77]. Post-transplantation, organoid grafts maintain a human epithelial barrier and develop lymphoid follicle-like structures over time. Moreover, when combined with an hHSC or BLT model, human IgA and IgG are produced in greater amounts in the local mucosa, and human dendritic cells are activated. Additionally, introducing human gut microbiota in such models is expected to enhance B-cell class switching in the gut mucosa and to strengthen local humoral immune responses [76, 77].

### Cytokine gene-introduced models (improved hHSC/hPBMC models)

Improved humanized mouse models have been developed by introducing human cytokine genes, primarily on NOG or NSG backgrounds, via KI or transgenic (Tg) approaches to express human hematopoietic and immunoregulatory factors [49]. These models can compensate for deficiencies in the number and function of human monocytes, dendritic cells, and NK cells, which are often suboptimal in conventional models, and thereby enhance antigen presentation-mediated adaptive immune responses. Representative improved models and their features are summarized as follows:

#### MITRG/MISTRG model (BALB/c × 129 background)

A model on a BALB/c × 129 background with human M-CSF, IL-3/GM-CSF, and TPO knocked in the MISTRG model additionally includes human SIRPα knock in.

This model is well suited for restoring the human myeloid immune axis, which is insufficiently reconstituted in conventional hPBMC or hHSC models [36, 78]. Although the proportion of hCD45⁺ cells in peripheral blood is largely comparable to that in standard hHSC models, the frequency of hCD33⁺ myeloid cells is significantly increased, and myeloid cells in non-lymphoid tissues such as the lung, liver, and colon have been reported to be ~ 10-fold higher than in hHSC models [36]. In addition, human dendritic cells exhibit mature antigen-presenting and cross-presentation capacity; consequently, both CD4⁺ and CD8⁺ T-cell responses are improved compared with conventional hHSC models, facilitating more human-relevant assessment of myeloid-T-cell interactions [36, 79]. In contrast, B-cell responses remain immature, making this model unsuitable for evaluating antibody responses and germinal center reactions [79]. Moreover, although neutrophils are clearly reconstituted and functional in the bone marrow, their frequency in peripheral blood has been reported to be lower than that in human peripheral blood [80]. In addition, MISTRG mice are prone to anemia, and the post-engraftment lifespan is approximately 10–12 weeks [36, 38]. Therefore, this model is useful for relatively short-term in vivo analyses of human monocytes/macrophages, dendritic cells, and neutrophils; for evaluating myeloid-dependent mechanisms of T-cell activation; and for modeling myeloid-mediated pathogenesis and assessing therapeutic efficacy. Indeed, it has been used to investigate human IL-6 trans-signaling in systemic sclerosis [78]. Notably, MISTRG is designed to express human cytokines physiologically from endogenous loci via a knock-in strategy, and is thus positioned as a model intended to mitigate cytokine overexpression artifacts compared with simple transgenic overexpression systems [81].

#### NSG-SGM3 model (NSG background)

An NSG-based model with transgenic expression of human SCF, GM-CSF, and IL-3 (the “SGM3” transgene) [82]. This model enables improved reconstitution of human myeloid immune cells compared with conventional hPBMC or hHSC models. Specifically, hCD33⁺ myeloid cells increase to approximately 3-fold higher levels than in standard hHSC models, and expansion of monocytes, dendritic cells, and mast cells enhances antigen presentation-driven T-cell responses [79, 83]. Accordingly, this model is well suited for analyzing myeloid–T-cell interactions that are difficult to capture in T-cell-skewed systems such as the hPBMC model [79, 83]. In contrast, transgenic expression of SCF, GM-CSF, and IL-3 has been associated with reduced stemness of CD34⁺ HSPCs and biased hematopoiesis due to short-term reconstitution [84, 85]. In addition, because human immunoglobulins, particularly the IgE subtype, tend to be produced, caution is warranted when evaluating humoral immunity under physiological conditions [82]. Therefore, while this model is useful for analyses of human myeloid compartments (including monocytes, dendritic cells, and mast cells), for investigating myeloid-dependent mechanisms of T-cell activation, and for studying IgE-related reactions and allergic inflammation, it is not necessarily optimal for long-term maintenance of hHSCs or for assessing physiological hematopoietic balance.

#### NOG-EXL model (NOG background)

A NOG-based model transgenic for human GM-CSF and IL-3 (“EXL”) [86, 87].

This model is particularly effective in restoring human type 2 inflammation and granulocyte-related immune cells that are poorly represented in conventional hPBMC or hHSC models [86, 87]. Reconstitution of eosinophils, basophils, mast cells, and dendritic cells is enhanced; after IL-33 administration, human IL-13 levels in Bronchoalveolar lavage fluid (BALF) increase significantly, and the lung exhibits increased hCD45⁺ cells, eosinophil infiltration, goblet cell metaplasia, and airway hyperresponsiveness [86]. In addition, systems using patient serum or antigens can reproduce human IgE-dependent passive cutaneous anaphylaxis, indicating functional recovery of human allergy/anti-parasite-related immune pathways that are otherwise difficult to recapitulate in hPBMC or hHSC models [86, 87]. Conversely, because this model drives GM-CSF/IL-3-dependent skewing toward myelopoiesis, particularly granulocytic differentiation, care is required when interpreting physiological hematopoietic balance and long-term immune reconstitution [86, 87]. Thus, this model is especially useful for analyses of human eosinophilic asthma, allergic inflammation, mast cell/basophil activation, and disease mechanisms or therapeutic efficacy targeting the IL-33/IL-13 axis [86–88].

#### NOG hFLT3L-Tg/mFlt3-null model (NOG background)

A NOG-based model overexpressing human Flt3 ligand and lacking mouse Flt3 [89].

This model is specialized for restoring the human dendritic cell axis, which remains insufficient in conventional hPBMC or hHSC engraftment models. In addition to major human dendritic cell subsets (cDC1, cDC2, and pDC), differentiation of Langerhans cells is also observed, enabling assessment of antigen presentation-driven T-cell activation, particularly cDC1-mediated cross-presentation to CD8⁺ T cells and cDC2-mediated induction of CD4⁺ T-cell responses [89, 90]. However, although dendritic cell subsets engraft prominently in the early phase after transplantation, they tend to markedly decline after 12 weeks, suggesting that the model may be less suitable for long-term analyses [89]. Therefore, this model is appropriate for short-term studies of human dendritic cell subset differentiation and function, for evaluating dendritic cell-dependent T-cell responses to vaccine or tumor antigens, and for dissecting cross-presentation mechanisms via cDC1 as well as dendritic cell networks including pDC [89, 90]. By contrast, when the primary objective is broad myeloid reconstitution or reconstruction of humoral immunity, other cytokine-introduced models such as MISTRG, NSG-SGM3, or NOG-EXL may be more suitable.

#### hIL-15-introduced models

Examples include SRG-15 (BALB/c × 129 background; human IL-15 KI), NSG-Tg (hIL-15) (NSG background; human IL-15 transgene), NOG-Tg(hIL-15) (NOG background; human IL-15 transgene), and NOG-IL-2/IL-15-Tg (NOG background; transgenic for human IL-2 and IL-15) [91, 92]. These models are primarily designed to restore human NK cells, which are limited in conventional hPBMC or hHSC models [91–94]. For example, NOG-Tg(hIL-15) can maintain peripheral blood-derived human NK cells for up to 3 months, which is markedly longer than in standard NOG mice, where such NK cells typically disappear within ~ 2 weeks [91]. In SRG-15, development and maintenance of tissue-resident NK cells, in addition to circulating NK cells, are promoted, and in NSG-Tg(hIL-15), generation and survival of functional HSC-derived human NK cells are also enhanced [93, 94]. Moreover, in NOG-IL-2/IL-15-Tg mice, human CD8⁺ T-cell activation is further augmented, potentially enabling the formation of a stronger cytotoxic immune response overall [91, 94]. In contrast, because these models are designed to strongly promote NK-cell persistence in an IL-15-dependent manner, they do not necessarily enhance NK-cell activity or homeostasis [91, 93, 94]. Indeed, in NOG-Tg(hIL-15), NK cells expanded in vivo may be quantitatively sufficient. Their cytotoxicity against K562 targets and IFN-γ production after stimulation are not necessarily high, suggesting that quantitative recovery and qualitative maturation may not coincide [91].

Additionally, in NOG FcγR-null/hIL-15-Tg mice (Fcγ receptor gene cluster knockout plus human IL-15-Tg), mouse ADCC/ADCP activity is virtually absent, enabling sensitive evaluation of ADCC effects mediated by human IgG antibodies and human NK cells [95].

Therefore, hIL-15-introduced models are useful for studying NK-cell-dependent cancer and infection immunity, for preclinical evaluation of NK-cell engagers and bispecific antibodies, and for assessing ADCC activity of Fc-dependent antibody therapeutics [92, 95, 96]. In terms of model selection, NOG-Tg(hIL-15) is suitable for long-term maintenance of peripheral blood-derived human NK cells; SRG-15 is appropriate for differentiation and analysis of NK cells including tissue-resident subsets; and NOG FcγR-null/hIL-15-Tg is advantageous for stringent evaluation of Fc-dependent effector functions.

#### hIL-6-introduced model (NOG background)

This model expresses human IL-6 transgenically on a NOG background and is characterized by restoration of immunosuppressive myeloid axes that are difficult to reconstitute in conventional hPBMC or hHSC models [84, 97]. In this model, differentiation of human monocytes/macrophages is promoted; HLA-DR-negative immature myeloid cells increase significantly in peripheral blood and spleen, and CD163-high tumor-associated macrophages (TAMs)-like cells are induced within tumors [97]. These cells express immunosuppressive molecules such as ARG-1, IL-10, and VEGF, and have been shown to suppress human T-cell proliferation under antigenic stimulation, thereby enabling a tumor immunosuppressive microenvironment to be modeled more readily than in hHSC models [84, 97]. Conversely, because IL-6-dependent skewing toward monocytes/macrophages and myeloid-derived suppressor cells (MDSC)-like cells is enhanced, this model is not suitable for assessing physiological multilineage hematopoiesis or balanced immune reconstitution [84, 98]. Accordingly, this model is useful for analyzing the differentiation and function of TAMs and MDSC-like cells and for evaluating mechanisms of tumor immune evasion mediated by myeloid immunosuppression [84, 97].

#### hIL-34-introduced model (NOG background)

This model introduces human IL-34 on a NOG background and is characterized by restoration of human CNS immune cells, particularly microglial cells, which are scarcely reconstituted in conventional hPBMC or hHSC models [99]. In this model, human microglia-like cells differentiate within the CNS and express microglia-associated molecules such as CX3CR1, CSF1R, TREM2, and P2RY12, facilitating analyses of human microglia-dependent chronic CNS infection and neuroinflammation that are otherwise difficult to address in standard models [99]. In addition, detection of HIV-1 RNA and p24 antigen in the brain suggests utility as a model of persistent CNS HIV infection [99]. However, these cells are microglia-like cells differentiated within the murine brain environment and do not fully recapitulate bona fide human brain-resident microglia. Moreover, reconstruction of the broader spectrum of CNS border-associated macrophages beyond the brain parenchyma remains difficult [99, 100]. Therefore, while this model is useful for analyses of chronic CNS infections such as HIV and microglia-dependent neuroinflammation, it has limitations in reproducing the full diversity of CNS macrophage populations and long-term neurodegeneration.

#### hIL-1β/IL-23-introduced model (NOG background)

A NOG-based model with transgenic expression of human IL-1β and IL-23 [101]. This model can amplify human Th17 inflammatory axes that are difficult to reproduce robustly in conventional hPBMC or hHSC engraftment models [101]. Following transfer of human CD4⁺ T cells, mice develop alopecia, weight loss, and T-cell infiltration in the skin earlier than in non-Tg controls. In the skin, pathogenic Th17 cells producing IL-17 and IFN-γ accumulate significantly, and marked infiltration of host mouse-derived neutrophils is also observed, supporting its utility for analyzing Th17-dominant human skin inflammatory pathways [101]. In contrast, this model represents an inflammation amplification system dependent on cutaneous GVHD, and certain pathological features, such as neutrophil infiltration, are partly driven by host mouse components; therefore, it does not reproduce the entirety of human psoriasis-like lesions [101, 102]. Accordingly, this model is suitable for analyzing the differentiation, tissue infiltration, and pathogenicity of human pathogenic Th17 cells and for evaluating interventions targeting the IL-23/IL-17 axis.

## Other models (including human gene-introduced models)

### c-kit mutant models (improved hHSC models)

The c-kit mutant models use hypomorphic c-kit mutations to impair endogenous mouse hematopoiesis and thereby facilitate hHSC engraftment. Consistent with this concept, the NBSGW model (a NOD-based c-kit mutant model) achieves high human chimerism without irradiation, reaching 97% in bone marrow and 61% in peripheral blood, with peripheral blood chimerism comparable to that of irradiated hHSC models [103]. The NOG-W/EXL model is generated by crossing the NOG-EXL model with a low-function c-kit mutant background [104]. Compared with the NOG-EXL model, it increases human CD45⁺ cells, B cells, and myeloid cells, particularly granulocytes and platelet/megakaryocyte lineage cells [104]. Its reported advantage, however, lies mainly in improved engraftment and lineage output. Therefore, this model is useful for functional analyses of human myeloid and B-cell compartments, particularly those focusing on granulocyte and megakaryocyte/platelet lineage development and on longer-term multilineage hematopoiesis [104]. The THX model is a more advanced c-kit mutant model generated by engrafting the NBSGW model with hHSCs, followed by 17β-estradiol conditioning [105]. Unlike conventional hPBMC or hHSC models, the THX model develops peripheral lymph nodes, Peyer’s patches, germinal center B cells, and Tfh cells, and mounts class-switched, hypermutated, and neutralizing antibody responses [105]. However, its limitations include reproducibility concerns related to the requirement for 17β-estradiol conditioning and limited human erythropoiesis. Accordingly, it is important to consider the use of the THX model in studies of germinal center reactions, antibody maturation, and vaccine-type humoral immunity, with these limitations in mind [105].

#### pRORγt-γc-Tg model (NOG background)

A NOG-based model (NOG pRORγt-γc-Tg) in which the mouse common γ-chain (Il2rγ, encoding the IL-7 receptor γ chain) is expressed under the RORγt promoter (critical for LTi cell differentiation) [106]. This partially restores mouse lymphoid tissue inducer (LTi) cells and promotes the formation of lymphoid structures akin to Peyer’s patches in the small intestine [106]. Consequently, germinal centers can form, and human IgA is produced in mucosal tissues, suggesting utility for analyzing mucosal vaccine responses and gut immune reactions.

### HLA gene-introduced models (improved hPBMC/hHSC models)

Humanized mouse models have also been developed by introducing human HLA genes, enabling the mice to express human antigen-presenting molecules [49, 107]. This addresses the issue that, in conventional models, human T cells trained on mouse MHC molecules cannot efficiently recognize human antigens, leading to weak antigen-specific responses [35]. In an HLA class I gene-introduced model (e.g., HLA-A*02:01), the humanized immune system can induce HLA-A2-restricted antigen-specific CD8⁺ T cells. For example, upon presentation of a specific antigen, human CD8⁺ T cells in these mice increase IFN-γ production, upregulate degranulation markers, and kill target cells in vivo, partially overcoming the weak T-cell responses due to MHC mismatch [108, 109]. Moreover, combining HLA class I expression with other modifications that boost antigen presentation (e.g., SGM3 or EXL models) or enhance effector functions of NK/CD8⁺ T cells (e.g., IL-15 introduction) further increases the numbers and functions of antigen-specific CD8⁺ T cells, leading to strong tumor or vaccine antigen responses [110, 111]. In an HLA class II gene-introduced model (e.g., HLA-DRB1*04:01), human DR4-restricted CD4⁺ T cells are positively selected and expanded, with increased Tfh-like cells in the periphery [112–114]. This is accompanied by an expansion of germinal center B cells and increased serum human IgG levels, indicating that B cell class switching, which is insufficient in class II-negative models, is enhanced. Furthermore, models that introduce both classes I and II concurrently exhibit both robust HLA-A2-restricted CD8⁺ T-cell responses and increased DR4-restricted Tfh cells, germinal centers, and IgG production [115, 116]. In summary, introducing an HLA class I gene alone primarily enhances cellular immunity (CD8⁺ T-cell responses); introducing a class II gene alone primarily enhances humoral immunity (CD4⁺ T-cell-mediated B-cell responses); and introducing both simultaneously strengthens both arms. Combined with human cytokine-expressing backgrounds, these models enable a more robust recreation of human immune responses.

### Overview of autoimmune disease models using humanized mice

Humanized mice can reproduce human-specific phenomena that cannot be observed in normal mice and enable the evaluation of therapeutics (e.g., human-specific antibodies) that act only on human cells. Accordingly, various disease models have been created based on humanized mouse models outlined above. In PBMC- or lymph node-transferred humanized mice, donor patient TCR/BCR repertoires are retained, demonstrating that using patient-derived cells can partially recreate the patient’s disease state. This feature is widely leveraged, and many disease models incorporate patient PBMCs or lymphoid cells to introduce patient-specific pathology. Meanwhile, hHSC models, which reconstruct diverse immune subsets, are frequently used with disease-inducing stimulation known from wild-type mouse models, applied to the humanized mouse (e.g., pristane injection for lupus nephritis). Although disease induction by antigen immunization has been reported, no humanized mouse model has yet consistently induced robust antigen-specific immune responses to exogenous antigens, and reproducibility remains an issue. In the following section, humanized mouse models of immune-mediated diseases are described, with a focus on autoimmune diseases. Key features of each disease model are summarized in Table 1.

**Table 1:**
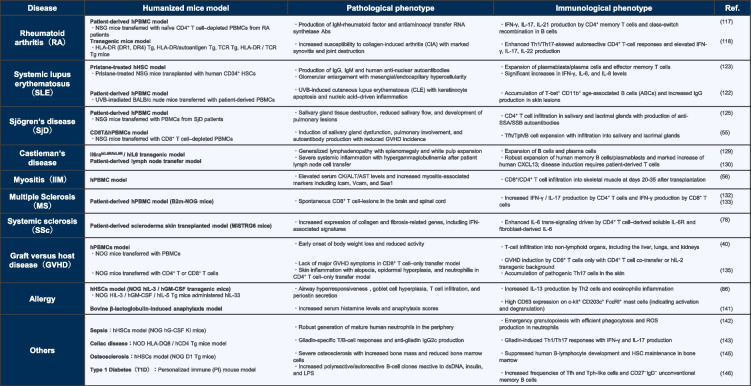
Various immune-related disease models based on humanized immune system models

Comprehensive summary of the methods used to create disease models, key phenotypes, and immunological characteristics of various immune-related diseases (RA, SLE, SjD, CD, IIM, MS, SSc, GVHD, allergy, and others) that can be developed based on humanized immune system models. *RA *rheumatoid arthritis, *SLE *systemic lupus erythematosus, *SjD *Sjögren’s disease, *IIM *idiopathic inflammatory myopathies, *MS *multiple sclerosis, *SSc *systemic sclerosis, *GVHD *graft-versus-host disease, *allergy *allergic disorders

### Rheumatoid arthritis (RA) model

Humanized mouse models for RA have evolved mainly for two purposes: (1) in vivo analysis of autoantibody production mechanisms by human autoreactive T and B cells and (2) preclinical evaluation of novel therapies involving the human immune system (such as cell therapies). In Ishikawa et al.’s method, NSG mice were sequentially engrafted with memory CD4⁺ T and B cells (after removing naive CD4⁺ T cells from hPBMCs), thereby achieving long-term engraftment of human T and B cells while avoiding lethal GVHD [117]. In this model, memory CD4⁺ T cells function as helper T cells in the spleen and lymph nodes, and B cells undergo class-switch recombination to produce IgM, IgG, and IgA [117]. Notably, using cells from patients with RA or patients with anti-aminoacyl tRNA synthetase syndrome, new IgM-type rheumatoid factor (RF) and anti-aminoacyl tRNA synthetase autoantibodies were induced in vivo and persisted long-term [117]. This model thus enables reproduction and analysis of T- and B-cell interactions and autoantibody production within a human immune system. Other approaches include transgenic models introducing RA-susceptible human HLA class II alleles (e.g., HLA-DRB1*04:01), human autoantigens, or autoimmune TCRs, and human-mouse chimeric models in which RA-derived synovial tissues, synovial fluid cells, hPBMCs, or CD34⁺ hHSCs are engrafted into immunodeficient mice [118]. These enable in vivo evaluation of how interactions between human immune cells and human synovial tissue lead to arthritis. Furthermore, in immunodeficient mice expressing an RA-susceptible HLA, transplantation of patient-derived HSCs and synovial grafts sharing that HLA, followed by infusion of patient-derived regulatory T cells (Tregs) or tolerogenic dendritic cells (tolDCs), has been tested to restore self-tolerance (suppressing autoimmune responses) and assess changes in autoantibody levels, synovial inflammation, and bone/cartilage destruction [118]. This serves as a model for evaluating immunotolerance therapies in the human immune system.

#### Reproducibility and limitations

In a humanized mouse model of rheumatoid arthritis, naïve CD4⁺ T cell-depleted human PBMCs are transferred into NSG mice, allowing long-term engraftment of human memory T and B cells with reduced lethality from GVHD [117]. Using PBMCs from patients with RA, B cells undergo class-switch recombination to produce IgM, IgG, and IgA, with sustained induction of autoantibodies such as rheumatoid factor. Engrafted cells are largely hCD45⁺ and HLA-DR⁺, and include cytokine-producing T cells (IL-21, IL-17, IFN-γ). In extended settings, the peripheral human immune compartment consists of approximately 80% T cells and 10% B cells [117]. Despite these immunological features, clear clinical arthritis with persistent joint swelling occurs only in a subset of mice, and joint histopathology is limited and difficult to distinguish from changes associated with chronic xenogeneic GVHD, which develops within several weeks and constrains the observation window. Independent replication of this model remains limited, although earlier studies demonstrated that transfer of in vitro-activated RA synovial T cells can induce severe arthritis, underscoring the importance of activated T cells in joint pathology [119].

From a translational perspective, PBMC-reconstituted RA models show pharmacological responsiveness to clinically effective therapies. In NSG mice engrafted with RA patient PBMCs, treatment with the anti-TNF-α antibody infliximab reduced joint swelling and human inflammatory cytokine levels, supporting the relevance of TNF-α blockade in a human immune context [120]. However, most mice fail to develop sustained synovial hyperplasia, pannus formation, or progressive cartilage and bone destruction. Accordingly, while these models are useful for short-term evaluation of human autoantibody production, T-cell activation, and selected biologic responses, they do not fully recapitulate the chronic, joint-destructive pathology of human RA and have limited utility for comprehensive assessment of disease-modifying antirheumatic drugs or joint-protective effects.

### Systemic lupus erythematosus (SLE)/lupus nephritis model

Two main approaches have been reported for SLE models in humanized mice. One involves transferring PBMCs from patients with SLE into immunodeficient mice to evaluate human T/B cell responses and autoantibody production over a relatively short term. The other consists of reconstituting a human immune system by transplanting human CD34⁺ hHSCs and then inducing autoimmunity by intraperitoneal pristane injection [121]. The former is suited to analyze early T-cell-driven autoimmune responses and autoantibody dynamics, whereas the latter more reliably recapitulates systemic disease (including lupus nephritis) with kidney pathology [121]. A variation of the PBMC transfer model is a cutaneous lupus (cutaneous lupus erythematosus, CLE) model [122]. In this system, humanized mice engrafted with immune cells from patients with active SLE receive ultraviolet B (UVB) irradiation, which induces lupus-like skin lesions (without systemic organ involvement). In these mice, the lesional skin accumulated age-associated B cells (ABCs), a T-bet⁺ CD11c⁺ B-cell subset that is highly responsive to IL-21 and coordinates with TLR7/9 signals to produce local IgG [122]. This indicates that in CLE, local autoantibody production by an ABC-centered B cell subset can drive skin inflammation [122]. Conversely, in the systemic model (hHSC engraftment with pristane treatment), NSG mice transplanted with hHSCs and then given a single pristane injection produced various human autoantibodies, including ANA, anti-dsDNA, anti-histone, anti-Sm, anti-SSA, and anti-RNP70 [123]. The kidneys developed mesangial proliferative glomerulonephritis with leukocyte infiltration, deposition of human IgG/IgM in glomeruli, and proteinuria, closely resembling lupus nephritis. In peripheral blood and lymphoid tissues, CD27⁺ memory B cells, CD27⁻IgD⁻ double-negative B cells, plasmablast/plasma cells, and effector memory T cells increased, alongside elevated human inflammatory cytokines (e.g., IFN-γ, IL-6) and a type I interferon signature (upregulation of IFN-inducible genes) [123]. In summary, key features of SLE, including human immune system-driven autoantibody production, tissue inflammation, and heightened type I IFN responses, were reproduced in a humanized mouse model.

#### Reproducibility and limitations

Reproducibility and limitations of humanized mouse models of SLE vary depending on the experimental approach. In the pristane-treated hHSC model, the engraftment success rate at 12 weeks post-transplantation, prior to pristane administration, was reported to reach 94% based on the proportion of mice achieving ≥ 10% hCD45⁺ cells in peripheral blood, with marked donor-dependent variability and wide inter-individual differences in blood hCD45⁺ levels [123]. After pristane administration, the number of hCD45⁺ cells declined by approximately 50% within 8 weeks, and 50% of the mice died by 13 weeks, limiting the use of this model for long-term observation [123]. Immune reconstitution is dominated by CD4⁺ T cells and B cells, whereas myeloid cell engraftment remains limited, and germinal center formation is insufficient [123]. In addition, NSG mice lack functional complement activity due to Hemolytic complement (Hc) gene deficiency, which prevents reproduction of complement-mediated tissue injury such as membrane attack complex formation in glomeruli, a key pathological feature of human lupus nephritis [123]. B-cell class switching and sustained IgG autoantibody production are therefore suboptimal. In the UVB-irradiated BALB/c nude PBMC model of cutaneous lupus, quantitative human immune cell engraftment has not been sufficiently validated, and inflammatory infiltrates are largely of murine origin, with no independent replication reported to date [122]. Taken together, current humanized SLE models are useful for short-term analysis of human immune activation and early autoimmune responses but have limited capacity to recapitulate chronic disease progression, stable humoral autoimmunity, and complement-dependent tissue injury.

### Sjögren’s disease (SjD) model

For SjD (updated terminology from “Sjögren’s syndrome,” per a 2025 review [124]), humanized mouse models have included the transfer of PBMCs from patients with SjD into mice and immunization with human salivary gland antigens. Young et al. reported that NSG mice engrafted with PBMCs from patients with SjD developed CD4⁺ T-cell-dominant infiltrates and inflammation in the salivary and lacrimal glands, accompanied by reduced saliva production and increased serum IFN-γ and IL-10 [125]. Yang et al. similarly found that NSG mice receiving PBMCs from patients with SjD showed lymphocytic infiltration in the submandibular gland, salivary hypofunction, production of SjD-associated autoantibodies (anti-SSA/SSB), and lung involvement, along with activation of pathways related to type I IFN, apoptosis, and necroptosis [126].

Additionally, an hHSC model immunized with an antigen from the human salivary gland A-253 cell line developed SjD-like features: reduced saliva output, elevated anti-SSA autoantibodies, salivary gland destruction, and increased Tfh and Th17 cells, with disease amelioration by methotrexate or IL-17 blockade, indicating the involvement of these cells [127].

However, these conventional models require patient samples or immunization with specific antigens, which limits their general utility. Additionally, none of these models could induce the recently highlighted Tph cell subset. A “CD8TΔhPBMC model” was established by depleting CD8⁺ T cells from the PBMCs used for engraftment [55]. In this model, removal of CD8⁺ T-cell suppression leads to robust expansion of Tfh/Tph cells and B cells, allowing, within 3–4 weeks, the development of Tph-dependent SjD-like symptoms. Mice exhibit reduced saliva volume, increased anti-SSA/SSB production, and Tph cell infiltration in salivary glands; these symptoms are ameliorated by tacrolimus [55]. Single-cell RNA sequencing analysis of salivary gland-infiltrating cells revealed an increased presence of tissue-damaging CD4 T-cell populations in the CD8TΔhPBMC model, including IL-21⁺CXCL13⁺IFN-γ⁺ Tph cells and Granzyme K⁺Granzyme H⁺ CD4 T cells, which actively infiltrated the salivary glands [55]. Notably, these populations expressed characteristic markers such as ICOS, TIGIT, and CD40LG, which are also prominently observed in the salivary glands of human patients. These findings demonstrate, through the application of advanced omics-based technologies, that the CD8TΔhPBMC model recapitulates key immunopathological features of the human disease [55]. Notably, GVHD is less severe than in the standard hPBMC model, enabling observation of SjD pathology without GVHD symptoms. Lung involvement with elevated surfactant protein D (SP-D, a lung injury marker) is also observed. Importantly, transfer of the expanded Tph cells from a CD8TΔhPBMC model into a new NSG mouse is sufficient to induce salivary gland destruction and hyposalivation, providing direct in vivo evidence of Tph-cell cytotoxicity, a rare demonstration of pathogenic function for this cell type [55]. Thus, humanized mouse models of SjD have evolved to recapitulate both glandular and extraglandular manifestations, with particularly meaningful implications that human-specific cell subsets (Tfh and Tph cells) drive disease. Simultaneously, these models still face challenges in accurately reflecting the contributions of immune components beyond CD4⁺ T cells, such as antigen-specific triggers or CD8⁺ T cells, and further improvements are anticipated.

#### Reproducibility and limitations

Humanized mouse models of Sjögren’s disease show moderate reproducibility with important model-specific limitations. In patient-derived PBMC transfer models, one study using PBMCs from four SjD patients and four healthy donors, with three NSG mice per donor, reported relatively low inter-mouse variability in salivary gland immune infiltration and salivary flow reduction [125]. Engrafted immune cells were predominantly CD4⁺ T cells, with an approximate CD4⁺:CD8⁺ ratio of 2:1, and similar findings, including Th17 infiltration and elevated type I interferon signatures, have been reported by other groups, supporting a certain degree of reproducibility [128]. However, in these PBMC-based models, humoral immune pathology is incompletely reproduced, as consistent production of disease-relevant autoantibodies and robust germinal center formation have not been demonstrated, limiting their utility for evaluating B-cell- or autoantibody-targeted therapies [125].

The CD8TΔhPBMC model was developed to address some of these limitations by depleting CD8⁺ T cells prior to PBMC transfer. In this model, GVHD severity is reduced and the observation window is extended to approximately 3–4 weeks, allowing reproducible expansion of Tfh and T peripheral helper (Tph) cells together with B cells, as well as induction of SjD-like features such as salivary hypofunction and anti-SSA/SSB autoantibody production [55]. Although donor-dependent variability in the magnitude of Tph, Tfh, and B-cell expansion has been observed, key phenotypes are consistently reproduced across donors. Nevertheless, disease development remains restricted to a relatively short time frame, and long-term chronic glandular destruction cannot be assessed. Overall, PBMC-based SjD models are most suitable for short-term evaluation of human T-cell-driven glandular inflammation, whereas the CD8ΔhPBMC model provides improved access to Tph/Tfh–B-cell interactions but still does not fully capture chronic, long-standing Sjögren’s disease pathology.

### Castleman disease model

Castleman disease is a lymphoproliferative disorder characterized by lymph node enlargement and systemic inflammation; among its forms, idiopathic multicentric Castleman disease (iMCD-NOS) is a refractory subtype that can progress to multiorgan failure due to an unknown inflammatory cause. Humanized mice have been used to investigate pathogenesis and test therapies. Ueda et al. generated an hIL-6R/hIL-6 mouse by crossing a human IL-6 receptor KI mouse (in which mouse Il6r was replaced with human IL6R cDNA) with a human IL-6 transgenic mouse, thereby creating a model driven by human IL-6 signaling [129]. This model displayed generalized lymphadenopathy, splenomegaly with increased B cells and plasma cells in the splenic white pulp, and extramedullary hematopoiesis (findings reminiscent of Castleman disease). Administration of tocilizumab (anti-human IL-6R neutralizing antibody) significantly reduced spleen weight and plasma cell counts and led to increases in serum soluble human IL-6R and human IL-6 levels consistent with clinical pharmacodynamics [129]. These results indicate that the model is suitable for evaluating the in vivo efficacy and pharmacodynamics of anti-human IL-6R therapeutics.

Separately, Harada et al. reported that an iMCD model could be achieved by transplanting a single-cell suspension of lymph node cells from patients with iMCD-NOS into NSG mice [130].

In mice engrafted with iMCD cells from patients, robust expansion of human memory B cells and plasmablasts occurred alongside the growth of CD45⁺ human hematopoietic cells, leading to dramatic elevations of human IgG, IgA, and IgE in serum and severe systemic inflammation. Strikingly, if human CD3⁺ T cells were removed from the inoculum, these pathological features did not develop, implicating patient-derived T cells as indispensable for disease pathogenesis [130]. Analysis of human CD4⁺ T cells in the spleen revealed a marked accumulation of Tph cells with a PD-1^high^CXCR5^−^CCR2^+^ phenotype, and a corresponding massive increase in serum human CXCL13. Treatment with an anti-human CXCL13-neutralizing antibody mitigated systemic inflammation and prolonged mouse survival, indicating that Tph cell-derived CXCL13 is a critical driver of iMCD pathology via B-cell activation and antibody production [130].

#### Reproducibility and limitations

Humanized mouse models of Castleman disease include both genetically engineered and patient-derived approaches. The human IL-6/IL-6R model reproducibly develops lymphadenopathy, splenomegaly, and plasma cell expansion and shows pharmacodynamic responses to IL-6R blockade, supporting its use for evaluation of IL-6-targeted therapies [129]. Similar lymphoproliferative phenotypes have been observed in related IL-6-driven models, although independent replication of the original model is limited. A major limitation is non-physiological amplification of IL-6 signaling and reliance on murine downstream components, which constrains mechanistic interpretation [129]. The patient-derived lymph node transfer model demonstrates a central role for T peripheral helper cells and CXCL13 in disease pathogenesis, with consistent induction of B-cell expansion and hypergammaglobulinemia, but its use is restricted by tissue availability and lack of external validation. Both models are limited to short observation periods and do not capture the chronic relapsing course of human idiopathic multicentric Castleman disease.

### Myositis (idiopathic inflammatory myopathy) model

Standard myositis models in wild-type mice involve immunization with muscle-specific antigens (e.g., C protein or TIF-γ) to induce muscle inflammation [131]. However, very few studies have focused on myositis in humanized mice, largely because models, such as hPBMC or hHSC mice, struggle to mount antigen-specific immune responses due to suboptimal antigen presentation. Recently, a standard hPBMC model spontaneously developed idiopathic inflammatory myopathy (IIM)-like symptoms, including GVHD, demonstrating its potential as a humanized model of myositis [131]. In this model, by days 20–35 post-engraftment, human CD8⁺ and CD4⁺ T cells infiltrated the muscle tissue, and serum levels of creatine kinase and AST were elevated, along with increased expression of genes, such as ICAM, VCAM, and Saa1, which are characteristic of IIM. Histopathology revealed muscle changes similar to those in patients with polymyositis (PM), and transcriptomic analysis showed that the model strongly mirrored PM pathology and partially recapitulated dermatomyositis (DM) features [131]. Significantly, depleting CD8⁺ T cells from the PBMC inoculum substantially improved the muscle symptoms, demonstrating that the pathology is CD8⁺ T-cell-dependent. Lung lesions often associated with IIM were also observed, and both muscle and lung symptoms were suppressed by prednisolone or tacrolimus, underscoring the model’s utility for drug efficacy evaluation [131]. In addition, RNA sequencing analysis of muscle tissue from this model mouse was performed to compare its gene expression profile with gene marker signatures characteristic of human IIM patients [131]. As a result, this model was found to exhibit gene expression patterns similar to those observed in PM and DM [131]. This analysis represents a good example of demonstrating the translational relevance of a disease model using transcriptomic profiling. These results indicate that the model closely parallels human disease and is suitable for testing therapeutics, including human-specific antibody drugs. Future challenges include developing a myositis model without GVHD interference and establishing models that reflect IIM subtypes, such as DM or inclusion body myositis. Achieving these will likely require reliably reproducing antigen-specific immune responses in humanized mice.

#### Reproducibility and limitations

In a PBMC-based humanized mouse model, IIM-like pathology develops reproducibly within 20–35 days after engraftment, characterized by infiltration of human CD8⁺ and CD4⁺ T cells into skeletal muscle, elevation of muscle injury markers, and transcriptomic similarity to human PM [131]. Disease severity is markedly reduced by CD8⁺ T-cell depletion, confirming CD8⁺ T-cell dependence, and muscle and lung inflammation respond to clinically used immunosuppressive agents [131]. However, disease onset coincides with xenogeneic GVHD, which restricts the observation period to approximately 4 to 5 weeks. Antigen-specific autoimmunity and myositis-associated autoantibody production are not reproduced, and validation across independent laboratories has not yet been reported. Therefore, this model is suitable for short-term analysis of human T-cell-mediated muscle injury but not for modeling chronic or autoantibody-driven IIM.

### Multiple sclerosis (MS) model

MS is an autoimmune demyelinating disease of the CNS. Conventional mouse models (e.g., experimental autoimmune encephalomyelitis, EAE) cannot capture human-specific immune backgrounds, which has been a limitation. Papazian et al. established a humanized MS model using B2m-knockout NOG mice (lacking mouse B, T, and NK cells, and MHC class I expression) and engrafting them with PBMCs from either a patient with MS or a healthy donor with known HLA-DRB1 haplotypes [132]. In mice engrafted with PBMCs from a patient with MS who was HLA-DRB1*15:01-positive, human T cells, especially CD8⁺ T cells, infiltrated the gray matter of the brain and spinal cord, forming spontaneous inflammatory lesions. Moreover, when an EAE-inducing immunization with a myelin antigen was added, lesions worsened, with infiltrates containing CD4⁺/CD8⁺ double-positive T cells. By contrast, mice engrafted with PBMCs from an HLA-DRB1*15:01 healthy donor or an MS patient with HLA-DRB1*13:01 did not develop significant lesions, indicating that the combination of an MS-risk HLA allele (DRB1*15:01) with patient-derived immune cells is crucial for CNS lesion formation [132]. Additionally, using the same B2m-null NOG/hPBMC model, Dagkönaki et al. analyzed how the presence or absence of the HLA-DRB1*15:01 allele and a history of Epstein-Barr virus (EBV) infection in donors affected immune reconstitution [133]. These studies demonstrate that in humanized mouse models of MS, patient-specific factors, such as HLA class II haplotypes and latent viral infections (e.g., EBV), can influence T-cell responses and the development of CNS inflammation.

#### Reproducibility and limitations

In humanized mouse models of MS, reproducibility is strongly donor- and HLA-dependent. In the B2m-deficient NOG hPBMC model, inflammatory CNS lesions developed in three of five mice engrafted with PBMCs from HLA-DRB1*15:01-positive, EBV-seropositive MS donors, whereas mice receiving PBMCs from DRB1*15:01-negative donors showed only minimal infiltration [132]. Similar HLA- and EBV-dependent trends have been reported in related studies, supporting partial reproducibility [134]. Although human T cells, particularly CD8⁺ T cells, infiltrate the central nervous system, extensive demyelination, axonal loss, or progressive neurological deficits are not observed [132]. In addition, B-cell responses, germinal center formation, and intrathecal IgG production remain insufficient, preventing reproduction of oligoclonal band formation and chronic B-cell-mediated pathology. Consequently, these models are suitable for studying HLA-dependent human T-cell infiltration into the CNS but are not appropriate for modeling chronic or progressive multiple sclerosis or for evaluating B-cell-targeted therapies.

#### Scleroderma/skin fibrosis model

Scleroderma (systemic sclerosis) is characterized by immune dysregulation and fibroblast activation leading to fibrosis of the skin and internal organs, with IL-6 signaling implicated in its pathogenesis. Odell et al. developed a model using MISTRG6 immunodeficient mice (bearing multiple knock-in alleles for human cytokines, specifically hM-CSF, hIL-3, hGM-CSF, hTPO, and hIL-6) engrafted with human hHSCs, and then transplanted either fibrotic skin from a scleroderma patient or healthy skin onto these mice [78]. In the MISTRG6 mice reconstituted with human immune cells, the grafted skin maintained human CD4⁺ and CD8⁺ T cells and human endothelial and perivascular cells over the long term, whereas mouse fibroblasts replaced the fibroblasts in the graft [78]. In control conditions without human immune reconstitution, the transplanted skin retained a scleroderma-like fibrosis signature: high expression of collagen and type I interferon-related genes, human T-cell activation, and elevated human IL-6 expression in fibroblasts. However, when hHSCs were transplanted (establishing a human immune system), these fibrosis-related gene expression and T-cell activation markers in the graft were reduced, and fibrosis was attenuated [78]. Mechanistic analysis revealed that soluble IL-6 receptor (sIL-6R) derived from human CD4⁺ T cells can form a complex with IL-6 produced by fibroblasts, activating gp130-mediated IL-6 trans-signaling that induces extracellular matrix gene expression [78]. The presence of a human immune system suppressed this pathway, revealing that interactions between immune cells and fibroblasts modulate fibrosis.

#### Reproducibility and limitations

The MISTRG6 skin graft model of systemic sclerosis shows limited reproducibility and technical constraints. Among neonatal MISTRG6 mice transplanted with hHSCs, only approximately 30% achieved sufficient immune reconstitution to allow subsequent transplantation and analysis of patient-derived skin grafts [78]. Although blood immune reconstitution is characterized by a high proportion of CD33⁺ myeloid cells and CD4⁺ T-cell-dominant lymphocytes, independent replication of this model has not yet been reported. A major limitation is that human fibroblasts within the transplanted skin are gradually replaced by murine fibroblasts, which precludes evaluation of human fibroblast-intrinsic fibrotic mechanisms and limits assessment of therapies directly targeting human extracellular matrix production [78]. As a result, this model is best suited for mechanistic studies of immune cell–skin interactions rather than for comprehensive preclinical evaluation of antifibrotic therapies.

#### GVHD model

Immunodeficient mice injected with hPBMCs to induce acute GVHD-like symptoms have long been used because they can recapitulate pathology with a small number of human lymphocytes. For instance, simply injecting hPBMCs intravenously into NOG mice (without conditioning) causes rapid weight loss and reduced activity, with human T-cell infiltration and tissue damage in multiple organs (skin, liver, lung, kidney) within a short time [40]. To analyze pathogenic T-cell subsets, models using sorted human T cells have been informative. Transferring human CD8⁺ T cells alone rarely induces severe GVHD, but co-transferring CD4⁺ T cells or using human IL-2 transgenic recipient mice leads to massive CD8⁺ T-cell expansion and severe GVHD [135]. Conversely, transferring CD4⁺ T cells alone primarily causes skin lesions (epidermal hyperplasia, alopecia, neutrophil infiltration), which show accumulation of Th17 cells and IL-17-dependent inflammation [135]. These findings indicate that CD4⁺ T cells help amplify the cytokine milieu to enhance CD8⁺ T-cell cytotoxicity and that in skin lesions, a Th17-IL-17 axis is a key pathogenic pathway. Moreover, in an allograft rejection model where human skin and spleen cells were transplanted into mice, administration of an IL-21R antibody improved graft survival, implicating IL-21 signaling in the GVHD response [136].

The hPBMC-induced GVHD model is also used in preclinical testing of cell therapies. For example, in NSG mice with acute GVHD induced by hPBMC transfer, intravenous infusion of human mesenchymal stem/stromal cells (MSCs) prolonged survival and ameliorated liver and small intestine lesions [137]. Specifically, IFN-γ-preactivated MSCs (the so-called MSC2 phenotype) demonstrated significant suppressive effects even when co-administered with hPBMCs, with in vivo evidence of reduced expansion of donor CD4⁺ T cells and lower serum TNF-α [137]. Thus, human GVHD models provide a valuable platform for directly evaluating the efficacy of cell therapies and biologics under conditions of human T-cell responses and cytokine production.

Findings from mouse allogeneic GVHD models have also informed the understanding of humanized GVHD. For example, tissue injury from conditioning and rapid donor T-cell activation/infiltration are central in acute GVHD, whereas B-cell responses and fibrosis with autoimmunity-like mechanisms are key in chronic GVHD [138]. In a chronic GVHD model with SLE-like features, when transferring MHC-mismatched spleen cells, removing host CD4⁺ T cells nearly abolished anti-dsDNA autoantibodies, nephritis, and mortality, whereas removing host CD8⁺ T cells did not affect the disease [139]. Additionally, if sufficient donor CD8⁺ T cells were present, autoantibody production and kidney injury were suppressed, indicating that cytotoxic CD8⁺ T cells can regulate the progression of chronic GVHD and SLE-like symptoms [140]. These observations illustrate how T-cell subset interactions influence GVHD outcomes, consistent with findings in humanized GVHD models.

#### Reproducibility and limitations

PBMC-induced xenogeneic GVHD models show highly reproducible human immune cell engraftment, with nearly all mice achieving approximately 50% hCD45⁺ cells in peripheral blood and more than 80% engraftment in major organs such as the spleen, liver, and lung, where T cells constitute about 90% of human leukocytes [40]. Despite this robustness, the pathological process is driven by human T cells recognizing murine MHC molecules rather than human HLA, which fundamentally differs from clinical allogeneic GVHD [40]. Myeloid cell contributions and complement-mediated injury are poorly represented, and the observation window is limited to approximately 6 weeks because of rapid disease progression and mortality. Therefore, while this model is well suited for short-term evaluation of acute human T-cell activation and immunosuppressive interventions, it is not appropriate for studying chronic GVHD or long-term fibrotic complications.

#### Allergic disease models

Humanized mouse models have also been used in allergic disease research to investigate pathogenesis and evaluate human-specific treatments [88]. In an asthma model, immunodeficient NOG mice transgenic for human IL-3/GM-CSF or IL-3/GM-CSF/IL-5 were engrafted with hHSCs to establish a mature human immune system. Subsequent intratracheal instillation of human IL-33 was used to trigger a human type 2 immune response [86]. This model demonstrated signs of human T-cell and mast cell infiltration in the airways, increased airway hyperresponsiveness, goblet cell hyperplasia, human IL-13 production, and resultant airway remodeling, recapitulating a type 2 inflammatory response resembling human asthma. In strains that also express IL-5, marked eosinophilia was induced, enabling modeling of eosinophilic airway inflammation. Furthermore, administration of an anti-human IL-13 antibody significantly suppressed airway inflammation and hyperreactivity, demonstrating the model’s utility for studying IL-33-IL-13-driven asthma and for evaluating anti-IL-13 therapy [86].

For food allergy or anaphylaxis, one approach is to engraft NOG-EXL mice (hGM-CSF/hIL-3 Tg) with hHSCs, passively sensitize them with a human IgE specific for an allergen (e.g., β-lactoglobulin (BLG) from cow’s milk), and then challenge them with that allergen [141]. After injecting BLG-specific human IgE and exposing the mice to BLG, they rapidly developed systemic anaphylaxis manifested by hypothermia and elevated serum histamine. These symptoms were abrogated by epinephrine, indicating that the model faithfully recapitulates human IgE-dependent immediate hypersensitivity reactions in vivo [141]. Moreover, in NOG-EXL mice engrafted with hHSCs, human IgM, IgG, IgA, and IgE (without deliberate sensitization) were spontaneously produced, along with long-term persistence of plasma cells and memory B cells [82]. This intrinsic human IgE production can be harnessed for passive systemic anaphylaxis testing, making it a valuable model for dissecting human IgE-producing mechanisms and evaluating IgE-mediated allergic responses.

#### Reproducibility and limitations

In the IL-33-driven asthma model using cytokine-transgenic humanized mice, immune reconstitution is relatively consistent, with CD4⁺ T cells predominating over CD8⁺ T cells in the lung and spleen and T cells accounting for the majority of leukocytes in BALF [86]. However, disease induction depends on exogenous IL-33 administration, and allergen-specific sensitization and natural allergen responses, such as those induced by house dust mite, are not reproduced. In addition, type 2 innate lymphoid cells remain scarce, indicating incomplete reconstruction of the type 2 inflammatory cascade [86]. In the IgE-mediated anaphylaxis model, disease incidence varies by challenge route, with approximately 58% of mice responding to oral β-lactoglobulin challenge and a higher response rate but increased mortality following intravenous challenge [141]. Intestinal inflammation and oral sensitization mechanisms are not modeled, and independent replication has not yet been reported. Accordingly, these allergy models are best suited for evaluating acute effector responses rather than chronic allergen-driven disease mechanisms.

### Other disease models (type 1 diabetes, sepsis, celiac disease, bone marrow niche, etc.)

In addition to the above, humanized mice have been employed in a variety of other disease models. In a sepsis model, a NOG-hG-CSF mouse (with the human G-CSF gene knocked into the mouse Csf3 locus) was engrafted with hHSCs to achieve high levels of human neutrophil engraftment [142]. This model showed a high frequency of peripheral blood cells resembling human neutrophils, allowing analysis of infection responses driven predominantly by human neutrophils and proving helpful in studying neutrophil-dependent sepsis.

For celiac disease, approaches using gliadin-sensitized mice or HLA-DQ8 transgenic mice have been described. Freitag et al. injected TIMP-GLIA (gliadin-encapsulated PLGA nanoparticles) intravenously into mice possessing gliadin-specific CD4⁺ T cells (either gliadin-sensitized models or HLA-DQ8 Tg mice) to test whether inducing antigen-specific oral tolerance could serve as a therapy [143]. Celiac disease is an HLA-DQ2/DQ8-restricted enteropathy provoked by gluten, characterized by gliadin-specific CD4⁺ T-cell responses, autoantibody production, and villous atrophy; a breakdown in antigen-specific tolerance underlies its pathology. TIMP-GLIA treatment suppressed proliferation and IFN-γ production of gliadin-specific CD4⁺ T cells, reduced anti-gliadin IgG titers, and alleviated intestinal inflammation preserving villi and body weight. In HLA-DQ8 Tg models, increased tolerance-related gene expression, including FOXP3, was observed, suggesting that the induction of antigen-specific immune tolerance improved disease [143]. These results highlight a potential new antigen-specific therapeutic strategy, such as oral tolerance induction for autoimmune enteropathy, demonstrated using models carrying human HLA.

Another example of intestinal inflammation involves a Th17-driven colitis model using PBMCs from patients with primary sclerosing cholangitis (PSC) and NSG mice. Zhu et al. employed an integrated approach combining high-dimensional flow cytometry, transcriptomic profiling, and metabolic analyses to delineate how the semi-synthetic bile acid 24-nor-ursodeoxycholic acid (NorUDCA) modulates Th17 cells in models of intestinal inflammation [144]. In addition to a classical CD4⁺ T-cell transfer colitis model, analyses using humanized NSG mice reconstituted with PSC patient-derived PBMCs demonstrated that NorUDCA suppresses pathogenic Th17 responses while simultaneously promoting their trans-differentiation into FOXP3⁺ regulatory T cells [144]. Mechanistically, integrated multi-omics analyses revealed that NorUDCA attenuates the glutamine-mTORC1-glycolysis signaling axis in Th17 cells, reducing inflammatory Th1-like Th17 cells and facilitating a shift toward a tolerogenic phenotype [144]. This study provides new insights into immune cell plasticity and therapeutic mechanisms and highlights how comprehensive omics integration substantially advances the understanding of humanized mouse models of intestinal inflammation [144].

For modeling bone sclerosis and altered marrow niches, a NOG-DLL1-Tg mouse (transgenic for human Delta-like 1) has been reported [145]. NOG-DLL1-Tg mice develop pronounced osteosclerosis (increased bone mass) and reduced bone marrow cellularity; when engrafted with hHSCs, these mice show impaired human HSC engraftment and B-cell development in the bone marrow [145]. This model evaluates how Notch signaling in the bone marrow niche and increased bone density affect hHSC maintenance and differentiation.

Regarding type 1 diabetes (T1D), a “personalized immune (PI) mouse” model was described, in which immunodeficient mice transplanted with HSCs from a T1D patient or a healthy donor reconstitute a human immune system [146]. In PI mice constructed with T1D patient HSCs, compared with those with healthy HSCs, there was an increase in PD-1^high^ICOS⁺ Tfh or Tph cells, an increase in CD27⁻IgD⁻ double-negative B cells, and a decrease in naive B cells. These findings reflect aspects of T- and B-cell interactions thought to be important in T1D pathogenesis, suggesting that recreating a patient’s immune system can help uncover disease-specific immune abnormalities [146].

For CNS autoimmune diseases, transgenic mice expressing disease-associated human HLA class II (such as HLA-DRB1*15:01 or DRB1*03:01) have been developed [147]. These HLA-transgenic models show that CD4⁺ T-cell responses to human myelin peptides and EAE susceptibility vary by HLA haplotype, contributing to the identification of disease-susceptibility genes, target antigens, epitopes, and B-cell involvement in MS and related diseases [147].

For pulmonary fibrosis, two types of models are described. In the human lung cell engraftment model, human lung cells are transplanted into immunodeficient mice such as NSG mice, enabling engraftment of mesenchymal cells, particularly fibroblasts. Moreover, in studies transplanting lung cells derived from patients with idiopathic pulmonary fibrosis (IPF), engraftment of disease-associated epithelial cells has also been reported. This model reflects the pro-fibrotic properties of patient-derived cells and induces persistent and irreversible lung remodeling [148, 149]. However, because it lacks a human immune system, it cannot recapitulate immune–stromal interactions that are central to chronic fibrotic lung diseases. Therefore, this model is useful for mechanistic analyses of human fibrotic effector cells and for evaluating therapeutics that directly target pathogenic fibroblasts and/or epithelial cells [148, 149]. In the human immune cell engraftment model, pulmonary fibrosis is induced by administering bleomycin to humanized mice generated using the hPBMC or hHSC model. This model captures inflammation- and immunity-related aspects of pulmonary fibrosis, including inflammatory cell infiltration, fibroblast activation, and collagen deposition [150]. Nevertheless, because the lung parenchymal cells are of murine origin and disease induction relies on bleomycin-mediated tissue injury, it cannot fully reproduce the chronic and spatially heterogeneous microenvironment characteristic of human pulmonary fibrosis. Accordingly, compared with the human lung cell engraftment model, it is better suited for fibrosis analyses under a human immune milieu and for preclinical evaluation of antifibrotic therapies, including human-specific biologics [150].

### Drug discovery applications of humanized mouse models

Humanized mouse models have been widely used at many stages of drug discovery to evaluate therapeutic candidates for immune‑mediated diseases. These models play a key role in revealing human‑specific disease mechanisms and in providing a scientific rationale for targeting pathogenic immune pathways.

In GVHD‑like models generated by engrafting immunodeficient mice with human PBMCs, treatment with standard T-cell‑targeted immunosuppressants such as calcineurin inhibitors including tacrolimus and cyclosporine effectively ameliorates disease. Similar efficacy is observed with human TNF-α blockade and co‑stimulation inhibition using agents such as etanercept and abatacept [46, 135, 137]. These results mirror the clinical efficacy of these therapies in GVHD and confirm that humanized models can recapitulate T-cell-mediated pathology and its pharmacological control.

Likewise, in a human CD4^+ ^T-cell-driven skin inflammation model with prominent Th17 responses, neutralization of human IL-17A with a specific antibody significantly suppressed skin lesions [135]. This finding validated the pathogenic role of IL-17 and supported IL-17A as a therapeutic target.

In RA, NSG mice engrafted with PBMCs from patients responded to the anti-TNF-α antibody infliximab with reduced joint swelling, decreased levels of human inflammatory cytokines such as TNF-α and IFN-γ, and attenuated tissue destruction [120]. This late preclinical efficacy model confirmed TNF-α blockade in a human immune context and was consistent with the established clinical success of anti-TNF-α therapy in rheumatoid arthritis [9, 10].

By contrast, in a human RA synovium-implanted SCID mouse model, CTLA4-Ig did not ameliorate T-cell-rich synovitis, whereas IL-17A neutralization was effective [151]. This difference likely reflects the absence of de novo human T cell priming in the synovium-SCID model, which limited the effect of abatacept, and highlights the pathological contribution of IL-17 in specific T-cell-driven rheumatoid arthritis subsets. Notably, abatacept is clinically effective by preventing new T cell activation, whereas IL-17A blockade has shown only modest benefits in selected patient populations.

In SLE, human hematopoietic-stem-cell-engrafted models have been applied at the target identification and mechanism validation stage. These models reproduce the hallmark type I interferon gene signature of the disease [123]. This provided a mechanistic rationale for interferon pathway inhibition, which was later validated clinically by the success of the anti-interferon-α-receptor antibody anifrolumab [152]. In contrast, therapeutic strategies targeting IL-6 or BAFF have shown only modest efficacy, reflecting the multifactorial nature of SLE.

In SjD, a humanized NSG model reconstituted with patient PBMCs and immunized with salivary gland antigens was used for pathway validation. Neutralization of IL-17A significantly reduced sialadenitis and improved saliva secretion in this model [127]. These results supported the development of IL-17-targeted therapies for SjD, which are now in early clinical trials, and demonstrated the ability of the model to recapitulate Th17- and B-cell-driven pathology.

In systemic sclerosis, advanced humanized models incorporating a human immune system and human skin grafts were used to investigate disease mechanisms. These studies identified IL-6 trans-signaling as a key driver of fibrosis [78]. This insight provided a rationale for repurposing IL-6 receptor blockade. Consistent with this prediction, the IL-6 receptor antibody tocilizumab showed clinical efficacy in slowing progressive lung fibrosis and was approved for systemic-sclerosis-associated interstitial lung disease [153].

In inflammatory bowel disease, humanized mouse models have been used at the translational pharmacology stage to capture heterogeneity in therapeutic responses. NSG mice engrafted with PBMCs from patients with ulcerative colitis demonstrated that responsiveness to anti-TNF-α therapy depended on the dominant helper T-cell phenotype. Only mice reconstituted with Th1- or Th2-skewed immune cells responded well to TNF-α blockade, indicating a potential path toward personalized treatment strategies [154].

By contrast, clinical trials of IL-17A neutralizing antibodies failed to improve Crohn’s disease and in some cases exacerbated disease activity [155]. In comparison, IL-12 and IL-23 p40 inhibitors such as ustekinumab translated successfully into clinical practice [156]. These findings underscore the importance of appropriate target selection, as IL-17 may play protective roles in gut mucosal immunity.

In acute GVHD-like models based on rapid transfer of human PBMCs, humanized mice have been widely used for preclinical efficacy testing of immunosuppressive therapies. These T cell-driven models respond to calcineurin inhibition as well as to TNF-α and co-stimulation blockade, in agreement with clinical experience in GVHD treatment [46]. However, the short observation window and strong xenogeneic immune responses limit their applicability for long-term therapeutic evaluation.

In allergic inflammation such as severe asthma, specialized humanized models supported by transgenic expression of human cytokines have been developed. These models reproduce type 2 immune responses dominated by Th2 cells and eosinophils and have confirmed the pathogenic roles of IL-5 and IL-13 [86]. These findings directly supported the development of IL-5-targeted therapies and IL-4-receptor blockade, both of which have demonstrated substantial clinical efficacy in severe eosinophilic asthma [157, 158].

Emerging immunomodulatory therapies are also being evaluated in humanized mouse models. Low-dose interleukin-2 therapy preferentially expands regulatory T cells and suppresses autoimmunity. In NSG models engrafted with human PBMCs or CD3-positive T cells, low-dose interleukin-2 increased regulatory T cell frequencies and mitigated xenogeneic GVHD [159, 160]. These findings are consistent with clinical observations from regulatory T cell-based approaches. Next-generation interleukin-2 variants designed to enhance regulatory T cell expansion while minimizing effector T cell activation have shown similar effects in human-stem-cell-engrafted models [161, 162]. Humanized mouse models have also been used to evaluate T-cell-engaging bispecific antibodies. These agents redirect cytotoxic T cells toward specific targets and have demonstrated strong efficacy in oncology, with increasing interest in autoimmune applications. In humanized mice lacking mouse major histocompatibility molecules and engrafted with human target tissues, bispecific antibodies induced robust target cell elimination through human T cell activation [163, 164].

Collectively, human PBMC- and human hematopoietic-stem-cell-engrafted mouse models represent versatile preclinical platforms for evaluating the efficacy and mechanisms of monoclonal antibodies, receptor fusion proteins, and other biologics for autoimmune and inflammatory diseases. By reflecting patient-specific immune heterogeneity, these models also inform personalized therapeutic strategies. Ongoing refinements aim to better capture the complexity of human immunity and disease pathology, thereby improving predictive power for emerging therapies such as low-dose immune modulators and T-cell-redirecting biologics.

### Framework for selecting the appropriate humanized mouse model

**Fig. 2:**
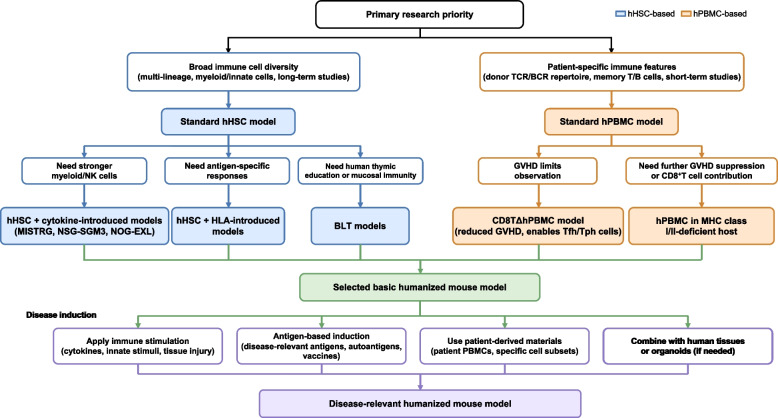
A simplified decision tree for selecting humanized mouse models. The first decision point is whether the research goal prioritizes broad immune cell diversity or patient-specific immune characteristics. Studies requiring long-term, multi-lineage immune reconstitution, especially involving myeloid or innate cells, should start with hHSC-based models. These models can be further refined using cytokine-introduced strains, HLA transgenes, or BLT approaches depending on experimental needs. In contrast, studies focusing on donor- or patient-specific immune repertoires and rapid T-cell responses should begin with hPBMC-based models, with CD8⁺ T-cell depletion or MHC-deficient hosts used to mitigate GVHD when longer observation or T–B cell interactions are required. After selecting an appropriate basic humanized mouse model, disease-relevant models can be generated by adding immune stimuli, disease-specific antigens, or patient-derived cells and tissues. This stepwise strategy separates model selection from disease induction, enabling flexible and purpose-driven construction of humanized disease models

Here, we integrate the basic humanized mouse platforms described above into a simple, practical decision framework. The primary considerations are (i) whether the study requires broad human immune cell diversity, particularly robust reconstitution of myeloid and other non‑lymphoid cells, or (ii) whether preserving donor- or patient‑specific immune features (such as TCR/BCR repertoires and immune memory) is more important. Additional decision axes include (iii) the required observation period (short‑term versus long‑term), (iv) tolerance for GVHD, and (v) the need for antigen‑specific, HLA‑restricted immune responses. These considerations are summarized as a conceptual decision tree in Fig. 2, which begins with two fundamental model pathways: hHSC‑based models and hPBMC‑based models.

### hHSC‑based models: prioritizing immune diversity and long‑term reconstitution

When a study requires multilineage human immune reconstitution and a long experimental window (months), an hHSC transplantation model is the appropriate starting point. Standard hHSC models provide stable, long‑term generation of human T cells, B cells, NK cells, and myeloid lineages with minimal GVHD, making them suitable for chronic disease modeling and sustained mechanistic analyses.

From this baseline, the model can be refined according to specific experimental needs. If enhanced representation or function of myeloid cells, dendritic cells, or NK cells is required, cytokine‑introduced hHSC models (e.g., MISTRG, NSG‑SGM3, or NOG‑EXL) can be selected to strengthen innate immune compartments. If the research question depends on antigen‑specific T‑cell responses or antibody production, introducing human HLA class I and/or class II genes enables HLA‑restricted immune responses that are weak in standard hHSC models. Alternatively, the BLT model, which incorporates human thymic tissue, can be used when proper human T‑cell education or mucosal immunity is particularly important. Overall, the hHSC‑based pathway offers a flexible platform for reconstructing a physiologically diverse human immune system and adjusting lineage composition or antigen recognition as needed.

### hPBMC‑based models: prioritizing patient specificity and rapid readouts

In contrast, when the primary goal is to capture patient‑specific immune characteristics or to obtain rapid in vivo readouts, hPBMC transfer models are more appropriate. Standard hPBMC models enable fast engraftment of mature human T cells within 1–2 weeks while preserving donor‑specific immune memory and autoreactive clones. This makes them well suited for short‑term analyses of human T‑cell function and personalized immune responses.

The main limitation of this approach is the rapid onset of xenogeneic GVHD, typically within 2–3 weeks. To extend the observation window and allow analysis of additional immune interactions, modified hPBMC models can be used. The CD8TΔhPBMC model reduces GVHD severity and enables expansion of CD4⁺ T cells and B cells, facilitating the study of Tfh/Tph–B-cell interactions and autoantibody production. If further GVHD suppression is required, hPBMC engraftment into MHC class I/II‑deficient host mice can markedly limit xenogeneic T‑cell activation and prolong cell persistence. These stepwise adaptations allow hPBMC‑based models to remain focused on donor‑specific immunity while partially overcoming their inherent temporal limitations.

### From basic model selection to disease‑relevant modeling

Once a suitable platform is chosen, disease‑relevant models are generated by applying additional experimental inputs, such as immune stimulation (e.g., cytokines or innate triggers), disease‑specific antigen immunization, or the use of patient‑derived materials (PBMCs, defined immune cell subsets, serum, or tissues). In summary, Fig. 2 provides a straightforward decision tree that guides readers from broad research priorities toward an appropriate basic humanized mouse model and its refinements.

## Conclusions and future perspectives

Humanized mouse models have become foundational tools that can recapitulate human biological systems and disease mechanisms that were previously difficult to study, and they are potent drivers of both basic research and drug discovery [165]. In particular, immune system-humanized mice demonstrate high utility not only for dissecting human immune mechanisms but also for disease modeling and PK/PD studies [166–168], with numerous innovative uses being proposed.

Even when analyzing tissue-infiltrating immune cells from organs with limited clinical samples, humanized mice offer considerable value despite interspecies differences. They have also helped study cells with human-specific biology (such as Tfh and Tph cells). Moreover, for evaluating human-specific therapeutics, especially antibody drugs, humanized mice are critical for elucidating mechanisms of action and gathering disease-relevant evidence that conventional models cannot.

Conversely, several challenges remain to be addressed. Chief among these is the difficulty of reliably and efficiently inducing antigen-specific immune responses to exogenous antigens such as vaccine responses or autoimmunity triggers in humanized mice, which likely limited the adoption of antigen-immunization models (e.g., collagen-induced arthritis). Stable lymphoid organ structures and functional antigen-presenting cell engraftment are necessary. Approaches, such as transplanting organoids that mimic lymphoid tissue or administering/overexpressing human cytokines are explored as solutions. If these hurdles are overcome, the impact on immunological disease research and vaccine development would be tremendous. Reproducing antigen-specific T- and B-cell responses in humanized mice could enable modeling the early initiation phases of autoantibody-driven diseases and lead to more appropriate disease models and therapeutic evaluations.

Furthermore, immune cell diversity and disease-specific features remain incompletely reproduced. The reconstitution of innate immune components, such as neutrophils and other myeloid cells, innate lymphoid cells, and even red blood cells, remains a significant challenge. Successfully incorporating these cells would deepen our understanding of inflammatory diseases and facilitate drug discovery for currently untreatable inflammatory conditions.

Further improvements are needed in humanizing host organs. As mentioned earlier, progress has been made in humanizing the liver, lung, bone marrow, and thymus; however, a broader range of tissues must be humanized and integrated to reproduce complex interactions between immune organs. This is directly linked to improving the accuracy of human PK/PD predictions for biologics. For example, introducing human FcRn into mice reproduces antibody recycling in humans and enhances the prediction of human antibody PK [168, 169]. Based on such advances, combining reconstruction of the human tissue environment with immune system reconstitution yields multifaceted humanized models capable of reproducing complex human pathologies that were previously unmodelable.

Looking ahead, the development of disease models will likely benefit from CRISPR genome editing and the use of patient-derived cells. Although many improvements have focused on the recipient mice, applying CRISPR edits to donor hPBMCs or hHSCs could enable functional evaluation of specific gene contributions in human immune cells to disease. Additionally, using patient-derived hPBMCs can help clarify the in vivo roles of patient-specific factors, such as autoreactive T cells or autoantibodies produced by patients’ B cells and plasma cells. Notably, using the CD8TΔhPBMC model, which avoids GVHD, one could maintain long-term tissue infiltration by Tfh/Tph and B cells along with sustained autoantibody production, thereby redefining their pathogenic roles in chronic autoimmune conditions.

In summary, advances in humanized mouse technology have opened new avenues for probing human disease pathology and human-specific immune mechanisms. These advances have contributed significantly to the nonclinical evaluation of human-specific therapeutics (such as antibody drugs). In the future, humanized mice are expected to play an increasingly important role in unraveling the complex mechanisms of immune diseases and developing next-generation therapies, especially antibody-based modalities, for those diseases.

## Data Availability

No datasets were generated or analysed during the current study.

## Abbreviations

ABC: Age-associated B cell
ACE2: Angiotensin-converting enzyme 2
ADCC: Antibody-dependent cellular cytotoxicity
ADCP: Antibody-dependent cellular phagocytosis
ALT: Alanine aminotransferase
ANA: Anti-nuclear antibody
APC: Antigen-presenting cell
ARG-1: Arginase 1
AST: Aspartate aminotransferase
BAFF: B cell-activating factor
BALF: Bronchoalveolar lavage fluid
B6RG: C57BL/6-Rag2-null IL2Rγ-null (immunodeficient mouse strain)
BCR: B cell receptor
BLT: Bone marrow-liver-thymus (humanized mouse model)
BRG: BALB/c-Rag2-null IL2Rγ-null (immunodeficient mouse strain)
CCR2: C-C motif chemokine receptor 2
CD: Cluster of differentiation (cell surface marker)
cDC: Conventional dendritic cell
c-kit: Mast/stem cell growth factor receptor
CIA: Collagen‑induced arthritis
CLE: Cutaneous lupus erythematosus
CNS: Central nervous system
CRISPR: Clustered regularly interspaced short palindromic repeats
CSF1R: Colony stimulating factor 1 receptor
CX3CR1: C-X3-C motif chemokine receptor 1
CXCL10: C-X-C motif chemokine ligand 10
CXCL13: C-X-C motif chemokine ligand 13
CXCR5: C-X-C chemokine receptor 5
DLL1: Delta-like ligand 1
DM: Dermatomyositis
dsDNA: Double-stranded DNA
DSS: Dextran sulfate sodium
EAE: Experimental autoimmune encephalomyelitis
EBV: Epstein-Barr virus
EpCAM: Epithelial cell adhesion molecule
FcγR: Fc gamma receptor
FcRn: Neonatal Fc receptor
FOXP3: Forkhead box P3
Flt3L: Fms-like tyrosine kinase 3 ligand
GM-CSF: Granulocyte-macrophage colony-stimulating factor
gp130: Glycoprotein 130 (IL-6 receptor signaling subunit)
GVHD: Graft-versus-host disease
Hc gene: Hemolytic complement gene
HCMV: Human cytomegalovirus
hHSC: Human hematopoietic stem cell
hPBMC: Human peripheral blood mononuclear cell
HIO: Human intestinal organoid
HIV: Human immunodeficiency virus
HLA: Human leukocyte antigen
IBM: Inclusion body myositis
ICAM: Intercellular adhesion molecule
IIM: Idiopathic inflammatory myopathy
IFN-α: Interferon-alpha (type I interferon)
IFN-β: Interferon-beta (type I interferon)
IFN-γ: Interferon-gamma (type II interferon)
IgA: Immunoglobulin A
IgE: Immunoglobulin E
IgG: Immunoglobulin G
IgM: Immunoglobulin M
IL: Interleukin
IL-2Rγ: Interleukin-2 receptor gamma chain
iMCD-NOS: Idiopathic multicentric Castleman disease, not otherwise specified
ILC: Innate lymphoid cell
IPF: Idiopathic pulmonary fibrosis
LTi: Lymphoid tissue inducer (cell)
JAK: Janus kinase
MASLD: Metabolic dysfunction-associated steatotic liver disease
NBSGW: Immunodeficient mouse model with mouse c-kit mutation
MDSC: Myeloid-derived suppressor cell
MHC: Major histocompatibility complex
MITRG: Immunodeficient mouse model with human M-CSF, IL-3/GM-CSF, TPO knock-ins
MISTRG: MITRG model with human SIRPα knock-in
MSC: Mesenchymal stem/stromal cell
NOD: Non-obese diabetic (mouse strain)
NOG: NOD/SCID IL2Rγ-null (immunodeficient mouse strain)
NOG-EXL: Immunodeficient mouse model with human GM-CSF and IL-3 transgenic expression
NOG-W/EXL: NOG-EXL with mouse c-kit mutation
NorUDCA: 24-Nor-ursodeoxycholic acid
NRG: NOD-Rag1-null IL2Rγ-null (immunodeficient mouse strain)
NSG: NOD/SCID IL2Rγ-null (immunodeficient mouse strain)
NSG-SGM3: Immunodeficient mouse model with human SCF, GM-CSF, and IL-3 transgenic expression
pDC: Plasmacytoid dendritic cell
P2RY12: Purinergic receptor P2Y12
PD-1: Programmed cell death protein 1
PK/PD: Pharmacokinetics/Pharmacodynamics
PM: Polymyositis
PSC: Primary sclerosing cholangitis
RA: Rheumatoid arthritis
RF: Rheumatoid factor
RNP: Ribonucleoprotein (e.g., anti-RNP autoantibody)
SAA1: Serum amyloid A1
SARS-CoV-2: Severe acute respiratory syndrome coronavirus 2
SCF: Stem cell factor
SCID: Severe combined immunodeficiency
SjD: Sjögren’s disease
SIRPα: Signal regulatory protein alpha
SLE: Systemic lupus erythematosus
SP-D: Surfactant protein D
SRG-15: Immunodeficient mouse model with human SIRPα and IL-15 knock-ins
SSA: Sjögren syndrome-related antigen A (Ro)
SSB: Sjögren syndrome-related antigen B (La)
TCE: T-cell engager
TCR: T cell receptor
Th: T helper (e.g., Th17 = IL-17-producing helper T cell)
Tfh: T follicular helper (T cell)
TKO: Triple knockout
TLR: Toll-like receptor
TNF-α: Tumor necrosis factor alpha
tolDC: Tolerogenic dendritic cell
Tph: T peripheral helper (T cell)
TPO: Thrombopoietin
Treg: Regulatory T cell
TREM2: Triggering receptor expressed on myeloid cells 2
TSLP: Thymic stromal lymphopoietin
UVB: Ultraviolet B
VCAM: Vascular cell adhesion molecule
VEGF: Vascular endothelial growth factor
